# Society Meetings

**Published:** 1912-01

**Authors:** 


### SOCIETY MEETINGS

The 90th annual meeting of the Medical Society of the County of Erie was held in the Buffalo Library Building on December 13, 1911, an unusually large number of members being present.

Treasurer, Dr. Albert T. Lytle, presented his annual report.

Preamble and resolution, in reference to appointment of a Surgeon General of the U. S. Public Health and Marine Hospital Service to succeed the late Dr. Walter Wyman, were presented by Dr. Lucien Howe.

Dr. H. J. Mulford, Chairman of the Committee on Necrology, submitted a brief report on the deaths of members during the year.

Dr. H. R. Hopkins, Chairman of the Committee on Public Health, submitted a report, illustrated by stereopticon, in which he directed attention to inadequate ventilation of public schools, antivaccination agitation and the necessity for combatting and suppressing same, typhoid fever prevalence in Buffalo, Niagara Falls, Lockport and the Tonawandas, resulting from the pollution of water supply by sewage.

Dr. C. A. Wall, Chairman of the Committee on Membership, recommended the election of Dr. Roy C. Fisher, 42 Lawrence Place and also of Dr. Joseph W. Young, 131 Allen Street, the latter transferred from the New York County Society. Both were elected to membership.

President Dr. Daniel McClure gave his annual address which was directed to be printed in the *Buffalo Medical Journal*. Incorporated in this motion was the following "that it is the sense of this society that no legislation pertaining to public health be considered by the State Legislature unless it emanates from the State Board of Health."

Report of the Board of Censors was presented by Chairman Dr. George L. Brown who gave the results of various prosecutions for violations of the medical laws and stated that several physicians are under indictment for alleged attempted abortion, and that the famous Porter Medical Co. has been driven from the city.

The Committee on Division of Fees was continued and requested to make a special report at the February meeting.

The election of officers resulted as follows:

President, Dr. Thomas H. McKee; First Vice-President, Dr. J. F. Whitwell; Second Vice-President, Dr. John V. Woodruff; Secretary, Dr. Franklin C. Gram; Treasurer, Dr. Albert T. Lytle; Censors, Drs. Irving W. Potter, Francis E. Fronczak, Arthur G. Bennett, Lawrence Hendee and John D. Bonnar; Chairman, Committee on Legislation, Dr. F. Park Lewis; Chairman, Committee on Public Health, Dr. Henry R. Hopkins; Chairman, Committee on Membership, Dr. Charles A. Wall; Delegate to the State Society, Drs. Albert T. Lytle, Julius Ullman, Julius Richter, J. F. Whitwell.

Retiring President McClure introduced President-elect McKee, who briefly thanked the members for the honor bestowed upon him.

---

### Chemung County Medical Society

THE annual meeting of the Chemung County Medical Society was held December 19, 1911, at the Society's rooms in the Federation Building, Elmira, N. Y.

The following officers for 1912 were elected: President, C. Haase (Buffalo '02), Elmira, N. Y.; Vice-President, Wm. Brady (Buffalo '02), Elmira, N. Y.; Secretary, C. F. Abbott, Elmira, N. Y.; Treasurer, G. V. R. Merrill, Elmira, N. Y.; Censors, H. W. Fudge (Niagara '93), Elmira, N. Y., R. G. Loop (Buffalo '97), Elmira, N. Y., S. Voorhees, Elmira, N. Y.; State Delegate, R. G. Loop, Alternate E. L. Bush, Horseheads (Buffalo, '02); VI District Delegate, H. W. Fudge, Alternate, C. Haase; Chairman Public Health Commission, A. H. Baker (Buffalo '85), Elmira, N. Y.; Chairman Commission on Legislation, R. P. Bush (Buffalo, '74).

MR. THEO. HORTON, Chief Engineer State Health Dept., addressed the Society on "Water Filtration." In the evening, he lectured before a public mass meeting under the Society's auspices at Convention Hall on "Sewage Disposal"—both lectures illustrated with stereopticon.

An amendment proposing to hold eight regular meetings of the society annually, instead of four as at present, was defeated.

An amendment was proposed increasing annual dues to $5.00 and rendering delinquent members ineligible for office. This will be voted on at the next annual meeting.

WM. BRADY,
Secretary.

The Buffalo Academy of Medicine will hold meetings as follows in January 1912, in the Public Library Building. The meetings begin at 8.30 P. M. (Physicians' standard time) and physicians visiting in Buffalo are cordially invited both to attend and to make themselves known.

January 2. (Surgery) Diagnosis of Intra-abdominal Conditions Presenting Acute Manifestations, Dr. Nathan Jacobson, Syracuse.

January 9. (Medicine) "Some Reasons why Incipient Pulmonary Tuberculosis is not Diagnosed," Dr. John H. Pryor, Buffalo.

January 16. (Obstetrics and Gynaecology) Conservative Surgery for Benign Tumors of the Uterus, Dr. Frederick Emil Neef, New York City.

January 23. (Pathology) Researches on Kidney Function, Dr. John T. Geraghty, Baltimore.

